# Supplementary material for: One test to rule them all: A qualitative study of formal, informal, and hidden curricula as drivers of USMLE “exam mania”
Source: PLoS One. 2023 Feb 3;18(2):e0279911. doi: 10.1371/journal.pone.0279911 (PMC9897523; doi:10.1371/journal.pone.0279911)
Supplement: S1 Appendix — Facilitator guide and guidelines for conducting focus groups of fourth-year medical students approaching the USMLE Step 2 CK exam. (DOCX) [file pone.0279911.s001.docx]

**S1 Appendix.** Focus Group Facilitator Guide

Welcome:

- Introduce self and assistant moderator (taking notes, providing technical support)

Our topic today is the Step 2 CK exam:

- You were selected because you have already taken Step 2 CK and volunteered to participate
- The results from today will be used in two ways: (1) to provide some feedback to

our administration about students’ experiences with this exam so that we can better

support you all, and (2) to share these experiences more broadly to give medical educators insight about this process.

Guidelines:

- No right or wrong answers, only differing points of view
- We’re tape recording, so please, if possible, only one person speaking at a time
- We’re on a first name basis – both for friendliness sake and to keep things anonymous
- You don’t need to agree with others, but you must listen respectfully
- My role as moderator will be to guide the discussion
- Talk to each other

I’d love to start with just an overall question: When you think of Step 2 CK, what comes to mind?

1. Some people say that performance on Step 1 influences your preparation and success on Step 2 CK. What do you all think about that?
2. What importance do you think is given to Step 2 CK by...?
   1. Residency directors?
   2. School administration?
   3. Students?
3. How do you think Step 2 CK compares to other factors considered in residency selection?
4. What advice did you all receive about when to take Step 2 CK?
   1. Who gave you this advice?
   2. Were there any discrepancies in the advice you received?
5. How did you all decide when to take Step 2 CK?
   1. What factors influenced this decision?
6. How did you prepare for Step 2 CK?
   1. How did this compare to how you prepared for Step 1?
   2. What was your study schedule like?
7. What kinds of support did you have throughout your preparation for Step 2 CK?
   1. What peer support did you have? (Other friends taking it at the same time, study groups, etc.)
   2. What institutional support did you have?
8. When you study for Step 1, everyone is generally studying at the same time. With Step 2 CK, your timing is more individualized. How do you think this affects you all?
   1. How do you think this impacts the class culture surrounding the exam?
9. What sorts of resources and support did you all receive for your preparation for Step 2 CK?
   1. How could these be improved?
10. How did you feel walking into test day?
    1. How does this differ to how you felt walking into Step 1?
    2. How prepared did you feel walking into Step 2 CK?
11. Now that you’ve taken the test, how do you feel about taking it when you did?
12. Is there anything else you would like to share with us about Step 2 CK?
