# Supplementary material for: One test to rule them all: A qualitative study of formal, informal, and hidden curricula as drivers of USMLE “exam mania”
Source: PLoS One. 2023 Feb 3;18(2):e0279911. doi: 10.1371/journal.pone.0279911 (PMC9897523; doi:10.1371/journal.pone.0279911)
Supplement: Attachment — Submitted filename: Response to Reviewers.docx [file pone.0279911.s002.docx]

**Explanation of Revisions**

| **Comment** | **Editor/Reviewers’ comment** | **Description of revisions** | **Location** |
| --- | --- | --- | --- |
| 1 | Academic Editor:  Please ensure that your manuscript meets PLOS ONE's style requirements, including those for file naming. | We have verified that our files meet PLOS ONE’s style requirements. | N/A |
| 2 | Academic Editor: Please provide additional details regarding participant consent. In the ethics statement in the Methods and online submission information, please ensure that you have specified (1) whether consent was informed and (2) what type you obtained (for instance, written or verbal, and if verbal, how it was documented and witnessed). If your study included minors, state whether you obtained consent from parents or guardians. If the need for consent was waived by the ethics committee, please include this information. | We have added this to our Methods section. | Pg. 7 |
| 3 | Academic Editor: Thank you for stating in your Funding Statement:  "This study was funded in part by the Association of American Medical Colleges (AAMC) Central Group on Educational Affairs (CGEA) Collaborative grant awarded to LEH."  Please provide an amended statement that declares *all* the funding or sources of support (whether external or internal to your organization) received during this study, as detailed online in our guide for authors at <http://journals.plos.org/plosone/s/submit-now>.  Please also include the statement “There was no additional external funding received for this study.” in your updated Funding Statement.  Please include your amended Funding Statement within your cover letter. We will change the online submission form on your behalf. | We have updated the funding statement within the manuscript and within our cover letter. | Cover letter, Manuscript pg. 20 |
| 4 | Academic Editor: Your ethics statement should only appear in the Methods section of your manuscript. If your ethics statement is written in any section besides the Methods, please delete it from any other section. | Done, we have removed the ethics statement in our Disclosures section and ensured it is only within the Methods section of the manuscript. | Pg. 7 |
| 5 | Academic Editor: Please include captions for your Supporting Information files at the end of your manuscript, and update any in-text citations to match accordingly. | We have added supporting information file captions at the end of the manuscript and have updated in-text citations. | Pg. 8, 28 |
| 6 | Academic Editor: Please review your reference list to ensure that it is complete and correct. If you have cited papers that have been retracted, please include the rationale for doing so in the manuscript text, or remove these references and replace them with relevant current references. Any changes to the reference list should be mentioned in the rebuttal letter that accompanies your revised manuscript. If you need to cite a retracted article, indicate the article’s retracted status in the References list and also include a citation and full reference for the retraction notice. | We have reviewed the reference list and ensure that it is complete and correct. | N/A |
| 7 | Reviewer #1: This is a well performed qualitative study on the impact of formal, informal and hidden curricula on the importance of step exams in medical school. this is a well thought out study and important. The authors formed focus groups of medical students to address the questions. the results show a great deal of stress associated with the exams. Medical schools should be cognizant of such data. | Thank you reviewer #1 for this positive feedback on our study. We agree that a broad population across medical education (including residency program directors, medical school faculty and administrators, and medical students themselves) would find our work highly relevant and could use this data as the foundation for improving the medical student experience. | N/A |
| 8 | Reviewer #2: This study is important at this time as with the transition to pass/fail, the Step 1 "mania' may transfer to Step 2CK. Thus it is high time that awareness be created about this issue. | Thank you reviewer #2 for this comment! We agree raising awareness is important, and a strength of our study is that we provide empirical, qualitative data to highlight this phenomenon. | N/A |
| 9 | Reviewer #2: It would be better if the authors explicitly state that this study was undertaken before the transition to pass/fail which took place in January, 2022 | This is a great suggestion. We have incorporated this explicitly into our Materials/Methods section. | Pg. 7 |
| 10 | Reviewer #2: The references are not appropriately edited according to PLOS One author guidelines. In the text, cite the reference number in square brackets. In the reference list at the end, do not use superscript. Go through other published work in PLOS ONE to get better accustomed to the reference formatting. | Thank you for this comment. We have corrected all reference formatting to be in accordance with PLOS One guidelines. | Pgs. 20-26 |
